# The optimal dose of Ramelteon for the better treatment adherence of delayed sleep–wake phase disorder: a dropout rate study

**DOI:** 10.3389/fneur.2023.1280131

**Published:** 2023-09-28

**Authors:** Shunsuke Takagi, Genichi Sugihara, Hidehiko Takahashi, Yuichi Inoue

**Affiliations:** ^1^Department of Psychiatry and Behavioral Sciences, Tokyo Medical and Dental University Graduate School, Tokyo, Japan; ^2^Yoyogi Sleep Disorder Center, Tokyo, Japan; ^3^Sleep Research Institute, Waseda University, Tokyo, Japan; ^4^Japan Somnology Center, Institute of Neuropsychiatry, Tokyo, Japan; ^5^Center for Brain Integration Research, Tokyo Medical and Dental University, Tokyo, Japan

**Keywords:** melatonin receptor agonist, delayed sleep–wake phase disorder, optimal dose, dropout rate, long-term treatment

## Abstract

**Background:**

Evidence regarding the effectiveness of melatonin receptor agonists in treating delayed sleep–wake phase disorder (DSWPD) remains limited. This study aimed to determine the optimal dose of ramelteon, a melatonin receptor agonist, for the better treatment adherence of DSWPD.

**Methods:**

The patients who were diagnosed definitely as having DSWPD by board-certified physicians specialized in sleep medicine and started to receive strategically timed ramelteon medications after the diagnosis were included. Data on the initial ramelteon dose and follow-up duration (up to 24 months) were collected retrospectively. Patients with treatment discontinuation, changes in ramelteon dose, or the addition of other sleep-related medications were considered dropouts. Kaplan–Meier estimates, log-rank tests, and Cox regression analyses were performed.

**Results:**

Overall, 373 patients were analyzed. The findings revealed that the 2 mg dose of ramelteon was associated with a lower dropout rate compared to the other doses (8 mg, 4 mg, and 1 mg). The dropout rate for the 2 mg group was estimated to have a hazard ratio (HR) of 0.5762 when compared with the 8 mg dose group. Sex did not reveal a significant HR, whereas older age exhibited a small but significant HR (0.9858).

**Conclusion:**

For achieving better adherence, a dosing regimen of strategically timed 2 mg ramelteon may be the best for the treatment of DSWPD. The therapeutic dose window for better adherence seems to center approximately 2 mg of ramelteon. Furthermore, caution should be exercised when treating younger patients to prevent dropouts.

## 1. Introduction

Delayed sleep–wake phase disorder (DSWPD) is characterized by difficulty falling asleep at the desired time and difficulty waking up at the required time (1). The disorder is prevalent in 4.3% of youth aged 15–30 years (2, 3), and approximately 10% of patients with chronic insomnia possibly have delayed sleep phase syndrome (4, 5). DSWPD is the most common circadian rhythm sleep–wake disorder that can significantly impact individuals’ quality of life (4–6). People with the disorder often suffer from extreme daytime sleepiness and tardiness, which can frequently cause poor achievement in their school life or workplace activities (5, 7). A delay in endogenous circadian timing of melatonin secretion or core body temperature rhythm is generally accepted as the intrinsic basis of the disorder (8–10). For DSWPD treatment, circadian phase advancement with melatonin or a melatonin receptor agonist has been recommended in addition to bright light therapy (9). However, most of the evidence for this treatment came from studies on healthy individuals rather than from those on patients with DSWPD, and whether patients with DSWPD will respond to the treatment with melatonergic drugs as do the healthy subjects remains unclear. Therefore, an optimal treatment strategy for patients with DSWPD must be explored.

Among melatonin receptor agonists, ramelteon, agomelatine, and tasimelteon have been commonly used for the treatment of DSWPD (4). These melatonergic drugs are administered as strategically timed treatment for the purpose of shifting the circadian phase of patients with DSWPD (4). Strategically timed treatment for the shifting circadian phase involves the scheduled administration of melatonergic drugs at a specific time of day in relation to dim light melatonin onset (DLMO), typically between 20:00 and 22:00 in the healthy population (4). This early-timing administration strategy is intended to correct the delay of endogenous melatonin rhythm in patients with DSWPD by providing exogenous melatonergic drugs depending on their phase response curve (11). The administration of oral melatonin receptor agonists at an earlier time referenced with DLMO is expected to mimic the normal endogenous melatonin secretion, advancing their circadian time toward a normal rhythm in the affected individuals (12). Previous studies have found that melatonin administered 5 to 7 h before baseline bedtime can advance the phase of the circadian rhythm in healthy participants (13). Since the time windows of advancement and delay of time shifting by melatonin receptor activation are closely attached and narrow (14), most of the previous trials with melatonergic drug administration failed owing to the time-shifting effect (14–16). Another important point of this strategy is to administer smaller doses of melatonin or melatonin receptor agonists for effectively shifting the circadian rhythm (13, 17, 18). Reportedly, as for ramelteon, the strongest DLMO time-shifting effects were observed with doses of 1, 2, and 4 mg, while the normally recommended dose of 8 mg (17, 19) for treating insomnia had no DLMO time-shifting effect. However, the optimal drug dose to exert a DLMO time-shifting effect has not yet been determined.

Long-term administration of exogenous melatonergic drugs is strongly recommended for the treatment of DSWPD (20, 21) because the risk of relapse after treatment cessation is as high as 90% (22–24). The chronic nature of DSWPD, which is characterized by a delay in the circadian timing of endogenous melatonin secretion unique to each individual’s circadian type and is linked to gene expression (25, 26), underlies the high risk of relapse (8, 9). However, despite the need for long-term treatment, there are currently no long-term studies on the treatment of DSWPD. Although a placebo-controlled double-blind study would provide the most robust evidence of treatment effectiveness, it can be laborious and costly. Alternatively, a dropout rate study may be a useful tool for assessing the effectiveness of long-term treatment, particularly in situations where treatment is frequently used in clinical practice. Given the high necessity of long-term treatment to avoid relapse of DSWPD, the dropout rate study reflects not only patient adherence but also the treatment effectiveness of ramelteon (27).

Herein, we conducted a retrospective dropout rate survey to determine the optimal ramelteon dose for the treatment of DSWPD.

## 2. Methods

This study was conducted at the Yoyogi Sleep Disorder Center in Tokyo, Japan, in accordance with the Declaration of Helsinki. The Ethics Committee of the Institute of Neuropsychiatry, Tokyo, Japan, approved the study and waived the need for informed consent from patients due to its retrospective nature.

### 2.1. Patients

We retrospectively investigated the data of consecutive patients with DSWPD who first visited the Yoyogi Sleep Disorder Center to seek treatment for sleep problems between January 2015 and December 2019. Patients aged <60 years who started receiving circadian phase-shifting treatment with ramelteon were eligible for this study. DSWPD diagnoses were established based on the International Classification of Sleep Disorders, Third Edition (ICSD-3) (1), by at least two board-certified sleep medicine psychiatrists. Diagnoses were confirmed through comprehensive interviews and sleep logs. The study was conducted between April and July 2022. Age at the first visit, sex, prescribed dose of ramelteon, other prescribed medications, length of the follow-up period, and treatment outcomes (follow-up loss, change in the dose of ramelteon, remission, etc.) were extracted from the patients’ medical records.

### 2.2. Ramelteon dosing

In this study, the majority of patients received a daily dose of 8 mg (one tablet), 4 mg, 2 mg, or 1 mg ramelteon in the speculated circadian phase-advancing zone (4). A small number of patients received a daily dose of 6 mg, and a few patients received a dose of less than 1 mg ramelteon. For the purpose of analyses, the patients receiving a daily dose of 6 mg were included in the 8 mg dose group and those receiving doses less than 1 mg in the 1 mg dose group.

### 2.3. Outcome measures

The primary outcome was treatment dropout from the respective ramelteon dose. Dropout was defined as the following: 1) cessation of ramelteon treatment for any reason, 2) changing the dose from the initially set dose, or 3) adding other sleep-related medications. The following cases were considered censored: 1) completion of 24 months of follow-up (28), 2) remission described in the medical records, or 3) transfer to other institutes to continue the same treatment.

### 2.4. Statistical analysis

For intergroup comparisons, one-way analysis of variance (ANOVA) was used for continuous variables, and Fisher’s exact test for multiple comparisons was used for nominal variables. Variables with significant differences in these analyses were subjected to post-hoc analyses; Tukey’s multiple comparison test was used for post-hoc analysis after one-way ANOVA, and Holm’s multiple comparison test was used for post-hoc analysis after Fisher’s exact test. For the inter-treatment group dropout analyses, Kaplan–Meier estimates were created, and the time until dropout was compared using the log-rank test. Cox regression analyses adjusted for demographic variables with significant differences (sex, age) were performed to calculate the hazard ratio (HR) with 95% confidence interval (CI) for the incidence of dropout. A two-tailed value of p of <0.05 was considered statistically significant. All statistical analyses except Fisher’s exact test and Holm’s multiple comparison analyses were performed using Prism 9 (GraphPad Software, Inc., La Jolla, CA, U.S.A.). Fisher’s exact test and Holm’s multiple comparisons were performed with R (29) using the RVAideMemoire package (30).

## 3. Results

### 3.1. Demographic data

We retrospectively investigated the data of 15,416 patients who visited the Yoyogi Sleep Disorder Center during the study period. Among them, 373 were identified as eligible for participation in this study (Table 1).

**Table 1:** Demographic data of the enrolled DSWPD patients.

Ramelteon nightly doses	8 mg	4 mg	2 mg	1 mg	Total
Total (*n*)	58	81	163	71	373
Follow-up completed	11 (19.0%)	16 (19.8%)	41 (25.2%)	3 (4.2%)	71 (19%)
Gender					
Female (*n*)	27	26	65	42	160
Male (*n*)	31	55	98	29	213
Age (years)	37.28 (11.56)	30.54 (10.50)	29.43 (9.38)	26.00 (10.85)	30.24 (10.78)
Medication period (mo)	4.8 (5.7)	6.1 (6.1)	7.1 (6.6)	5.6 (5.7)	6.2 (6.2)

This table shows the demographic data and variable differences (age and sex) for each ramelteon dose group. Ramelteon was administered at daily doses of 8, 4, 2, and 1 mg. The demographic variables included patient age and sex. Age and medication length were shown as ‘mean (standard deviation)’. Age was significantly different when ANOVA was used (*F* = 13.46, *p* < 0.001). Sex also showed a significant difference according to the Fisher’s exact test (*p* = 0.006). *p* (adjusted): value of *p* by Tukey’s multiple comparison test for age and Holm’s correction for multiple comparisons of sex.

f, female, m, male, mo, month, *n*, number.

The demographic data of the 373 patients (female: 160, male: 213; average age at first visit: 30.2 years) are presented in Table 1. Two patients who received 6 mg dose were included in the 8 mg group (3.4% of the group), and six patients who received less than 1 mg dose in the 1 mg group (8.4% of the group). Overall, 71 (11 (19.0%), 16 (19.8%), 41 (25.2%), and 3 (4.2%) patients in the 8, 4, 2, and 1 mg dose groups, respectively) patients (19.0%) completed 24 months of follow-up. In all the 302 patients, after excluding 24 months, the average length of follow-up was 6.2 (6.2) months (mean (standard deviation)). The lengths of the duration before dropout in patients of the 8, 4, 2, and 1 mg treatment groups were 4.8 (5.7), 6.1 (6.1), 7.1 (6.6), and 5.6 (5.7) months (mean (standard deviation)), respectively, after completion of the 24-month period. Furthermore, 111 (29.8%) patients continued to visit our clinic seeking any kind of treatment for the disorder, and the remaining 262 (70.2%) patients stopped visiting within 24 months of the first visit. Among the patients who stopped visiting, 13 achieved remissions, and 22 were transferred to other institutes.

Multiple comparisons among the ramelteon dose groups for demographic variables (age and sex) are summarized in Table 1. Age and sex revealed significant intergroup differences (*p* < 0.001 and 0.006, respectively). Post-hoc analyses revealed that the 4, 2, and 1 mg groups were significantly younger (p < 0.001, p < 0.001, and p < 0.001, respectively) than the 8 mg group, and the 1 mg group was significantly younger than the 4 mg group (*p* = 0.034). The 1 mg group included a significantly larger number of females than the 4 mg and 2 mg groups (*p* = 0.006 and *p* = 0.035, respectively).

### 3.2. Dropout rate analyses

**Figure 1:**
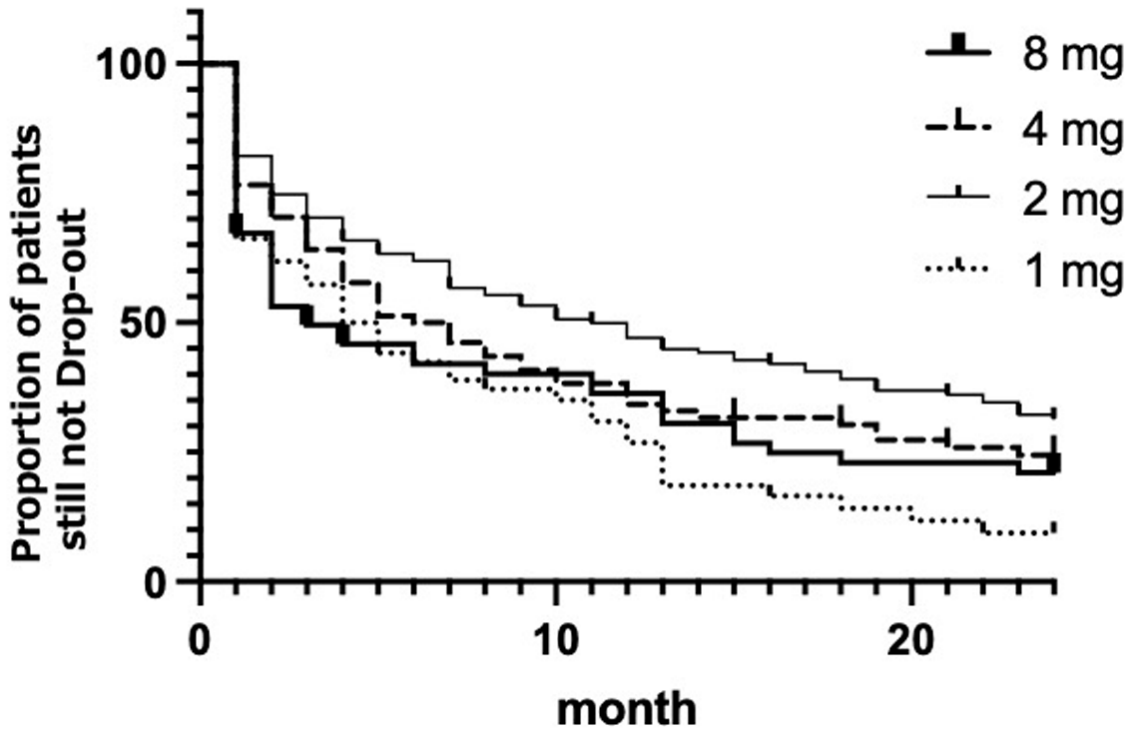
Kaplan–Meier plot for the each ramelteon dose. The x-axis represents the duration in months since the start of ramelteon treatment, and the y-axis denotes the estimated probability of survival. Thick line represents 8 mg of ramelteon; the dashed line represents 4 mg. The thin line represents 2 mg. Dotted line represents 1 mg.

The Kaplan–Meier plot for the dropout rate among the respective ramelteon treatment groups is shown in Figure 1. The 2 mg group showed the lowest dropout rate, and the highest dropout rate was seen in the 1 mg group. The log-rank test revealed a significant difference in the dropout rate between the treatment groups (χ2 = 14.45, df = 3, *p* = 0.0024).

The results of the Cox regression analysis of the dropout rate after adjusting for demographic variables are presented in Table 2. Sex was not a significant factor (*p* = 0.432). Meanwhile, the effect of age was significant (*p* = 0.022); older age was associated with a lower likelihood of dropping out. However, the effect of age was minimal, with an adjusted HR of 0.9858. Regarding the trend for dropout among nightly doses of ramelteon with the normally recommended dose of 8 mg as a reference, the only significant difference was found in the 2 mg dose group (*p* = 0.003), which showed a significantly lower dropout rate than the 8 mg group, with an adjusted HR of 0.5762.

**Table 2:** Hazard ratio of respective variables for dropout from the start of ramelteon treatment on DSWPD.

Variable	Adjusted hazard ratio	95% CI	*p*-value
Gender (*vs* f)			
Male	1.105	0.8620–1.421	0.432
Age (y.o.)	0.9858	0.9738–0.9978	0.022*
Ramelteon daily doses (reference; 8 mg)			
4 mg	0.7668	0.5160–1.148	0.192
2 mg	0.5762	0.4021–0.8391	0.003*
1 mg	1.024	0.6740–1.566	0.911

DSWPD: delayed sleep–wake phase disorder, *p*: value of *p*, f: female, m: male, y.o.: years old.

* statistical significance.

## 4. Discussion

To the best of our knowledge, this study has the largest sample size for evaluating the outcomes of pharmaceutical monotherapy for DSWPD. In this study, we targeted long-term adherence to ramelteon, the most widely used drug for treating DSWPD in Japan. In our study, more than 70% of patients stopped visiting our institute before the end of 24 months follow-up, and more than 80% did not complete the treatment with the initial dose of ramelteon. This result is in line with the previously reported relatively poor response rate of DSWPD treatment with melatonin (31). However, the Kaplan–Meier plot showed that the lowest dropout rate was seen in the 2 mg group, and the log-rank test found a significant difference in the dropout rate among the treatment groups. Cox regression analysis found a clear difference for the 2 mg dose group, which had a significantly lower dropout rate than the 8 mg group, with an adjusted hazard ratio of 0.5762. The results of the dropout rate analyses indicated that the optimal nightly dose of ramelteon for the strategically timed administration of DSWPD was 2 mg.

The key finding of this study was that the 2 mg dose of ramelteon, which is quite small but not the smallest, was found to be the most preferred dose with regard to dropout rates among DSWPD patients taking doses of 8, 4, 2, and 1 mg, respectively. Although it has been suggested that a relatively small dose of melatonin receptor agonists is suitable for advancing the sleep–wake phase in patients with DSWPD, the optimal dose has not been identified. Only a limited number of studies have compared the differences in the time-shifting effects among various doses of melatonin receptor agonists. Furthermore, as mentioned above, most of the evidence for this treatment comes from studies on healthy subjects rather than from those on patients with DSWPD; therefore, whether patients with DSWPD will respond to treatment with melatonergic drugs as would the healthy individuals remains undetermined. Since a dropout rate study is useful for assessing the effectiveness of long-term treatment of chronic diseases by estimating treatment adherence (32), dropout rate analyses of strategically timed ramelteon treatment in DSWPD would provide information on optimal dosing methods.

The reason why 2 mg ramelteon demonstrated better adherence can be partially explained by the time window of melatonin receptor stimulation. The stimulation of melatonin receptors may cause both advancement and delay of the circadian phase (14). The time-shift effect of melatonin receptor stimulation differs between time zones. The time zone in which the circadian rhythm can be advanced by the stimulation of melatonin receptors ranges between 7 h before and 2 h after sleep onset time (14). Thereafter, the time zone in which the circadian rhythm can be delayed follows. Therefore, receptor stimulation should be diminished within at least 2 h after sleep onset (33). Ramelteon has strong melatonin receptor potency (x 16.93 potency to melatonin) (34) and a slightly longer elimination half-life (2.6 h) (35) than melatonin. If the action of ramelteon continues through a longer time zone due to the administration of a higher dose of the drug, the melatonin receptor can be stimulated not only in the circadian phase advance time zone but also in the delay time zone (14, 18). This reciprocal action may attenuate phase shifting with ramelteon. Given this, the administration of 2 mg, a relatively low dose, of ramelteon in the phase-advancing time zone could be suitable for correcting the sleep–wake phase of DSWPD. In contrast, in this study, the lowest dose of ramelteon (1 mg) resulted in a lower adherence rate. The reason for this phenomenon is unclear; however, it is possible that a small dose window exists for the adequate treatment of patients with DSWPD.

Surprisingly, factors other than the dose had minimal influence on the dropout/adherence rate of ramelteon treatment for DSWPD. The HR for sex was not statistically significant. However, although the effect size was very small, age was significantly associated with the dropout rate, suggesting that older patients were less likely to dropout. Given this, we should be a little bit more cautious about young patients dropping out after treatment with ramelteon.

This study had several limitations. First, this study only evaluated the dropout rate, which evaluates treatment adherence. Further research is needed to evaluate other outcomes, including overall improvements in sleep quality and daytime functioning. Second, this study considered dropouts if the ramelteon dose was changed once. This may have overestimated the dropout rate for the first dose. Third, this study focused on only one specific melatonin agonist; therefore, the results cannot be generalized to other melatonergic drugs. Therefore, further research is needed to examine the optimal dosing strategy for melatonin receptor agonists in the treatment of DSWPD and to understand the safety and long-term effects of these medications. Fourth, the reasons for dropping out were not clarified. Some patients may have dropped out for reasons other than dosing, such as inappropriate time scheduling of medication. Fifth, the present study results might have been confounded due to comorbid conditions, such as mood disorders and developmental disorders (36). Although the information on comorbidities was checked before treating DSWPD by each attending physician, there might have been uncovered comorbidities. Sixth, this study exclusively targeted the initial ramelteon dose due to the diversity of reasons underlying medication dosage changes. Future inquiries should focus on subsequent adherence, post-comprehension of factors leading to ramelteon dosage adjustments. Seventh, the dropout rates among the different dosage groups, while statistically significant, did not exhibit substantial differences. DSWPD is known to be a condition with a low remission rate and a tendency for high dropout rates during treatment. Consequently, our study was influenced by this characteristic of DSWPD, resulting in relatively minor distinctions between the dosage groups.

The results of the study revealed that a 2 mg dose of ramelteon was associated with a lower dropout rate, suggesting that this dose may be more adherent in treating DSWPD than other doses. Therefore, strategically timed administration of 2 mg dose of ramelteon can be recommended for the treatment of DSWPD. However, younger patients need to be treated with increased care. This study suggests an optimum dose of ramelteon for the treatment of DSWPD. Further prospective studies, including placebo-controlled double-blind trials, are warranted to confirm our findings.

## Data availability statement

The raw data supporting the conclusions of this article will be made available by the authors, without undue reservation.

## Ethics statement

The studies involving humans were approved by the Institute of Neuropsychiatry. The studies were conducted in accordance with the local legislation and institutional requirements. The participants provided their written informed consent to participate in this study.

## Author contributions

ST: Conceptualization, Data curation, Formal analysis, Funding acquisition, Investigation, Methodology, Writing – original draft, Writing – review & editing. GS: Investigation, Methodology, Writing – original draft, Writing – review & editing. HT: Conceptualization, Data curation, Methodology, Supervision, Writing – review & editing. YI: Conceptualization, Data curation, Formal analysis, Funding acquisition, Investigation, Methodology, Supervision, Writing – original draft, Writing – review & editing.
